# Effects of Subconjunctival Bevacizumab and Ranibizumab on Corneal and Systemic Oxidative Stress Biomarkers in an Alkali Injury Model

**DOI:** 10.3390/life16030488

**Published:** 2026-03-17

**Authors:** Abdulhekim Yarbağ, Ebru Bardaş Özkan, Mustafa Ulaş, Yusuf Kemal Arslan

**Affiliations:** 1 Ophthalmology Department, Sakarya Sadıka Sabancı State Hospital, Sakarya 54580, Turkey; sadikadh.iletisim@saglik.gov.tr; 2 Department of Physiology, Faculty of Medicine, Erzincan Binali Yıldırım University, Erzincan 24100, Turkey; 3 Department of Physiology, Faculty of Medicine, Samsun University, Samsun 55100, Turkey; mustafa.ulas@samsun.edu.tr; 4 Department of Biostatistics, Faculty of Medicine, Çukurova University, Adana 01100, Turkey; ykarslan@cu.edu.tr

**Keywords:** ranibizumab, bevacizumab, corneal neovascularisation, oxidative stress, TAS, TOS, VEGF, alkali burn, rabbit model

## Abstract

The study tests the efficacy of subconjunctival ranibizumab and bevacizumab treatments in determining the oxidant and antioxidant levels during alkali-induced corneal neovascularisation. The researchers assigned 24 New Zealand White rabbits into four different groups, which included a healthy control, an alkali-injured control, a BV-treated group, and an RN-treated group, with six rabbits per group. All animals received alkali injury treatment except for the HC group. The AC group received six doses of subconjunctival saline 24 h after their injury, while the BV group and RN group received one dose of 0.5 mg of their respective treatment. The researchers conducted 14-day assays on normal tissue, blood plasma, and erythrocytes to determine total antioxidant status and total oxidant status. The researchers performed biochemical assays for total antioxidant status TAS and total oxidant status TOS on samples that were collected after the 14-day observation period. The corneal TOS levels increased in all injured groups when matched against the HC group. The RN group showed the highest corneal TOS levels, but the difference between the RN and AC and BV groups did not reach statistical significance. The injured groups showed higher corneal TAS levels than HC (*p* = 0.002), but the AC, BV, and RN groups showed no differences. The injured groups showed a significant decrease in erythrocyte TAS compared with HC (*p* < 0.001), but the injured groups showed lower plasma TOS levels than HC (*p* < 0.001). The researchers found no other systemic differences between the groups that received anti-VEGF treatment. The study results show that alkali injury leads to both local and systemic changes in redox status. The two anti-VEGF agents caused numerical changes in corneal oxidative parameters, but these changes did not achieve statistical significance. The research requires further investigation to understand potential agent-related effects on corneal redox balance, which should take place in larger, more detailed studies.

## 1. Introduction

The medical condition known as corneal neovascularisation (CNV) occurs when new blood vessels grow from the limbal vascular plexus into the corneal stroma, which normally lacks blood vessels. This condition causes corneal opacity and visual impairment, and it raises the probability of graft rejection [1]. CNV emerges because of different types of inflammation and infection, traumatic events, and chemical damage [2]. The condition presents an important challenge that ophthalmologists need to solve. The main molecular mediator that controls CNV development is vascular endothelial growth factor (VEGF) because it stimulates endothelial cell growth and movement and blood vessel leakage [3]. The study has shown that corneal injury leads to increased VEGF expression both in experimental research and clinical studies [4]. Therefore, current treatment protocols for anti-neovascular therapy regard VEGF inhibition as the most essential treatment method [5]. The ophthalmic field now uses anti-VEGF agents extensively to treat both posterior segment diseases and anterior segment conditions, which include CNV [6]. The most researched and used agents in clinical practice are bevacizumab and ranibizumab because their pharmacological profiles have been well-studied and they effectively reduce abnormal blood vessel growth [7,8].

Bevacizumab is a complete humanised monoclonal antibody that binds to all VEGF-A protein variants, while ranibizumab serves as a humanised antibody fragment that uses its recombinant design to achieve better tissue distribution and faster body clearance [9,10]. The distinctive structural attributes, together with their different pharmacokinetic profiles, will determine how each compound functions as an antiangiogenic treatment while producing various biological effects in the body that affect redox balance and oxidative stress control [1,11].

Oxidative stress has become a vital factor that causes corneal damage and leads to new blood vessel formation by damaging endothelial cells and triggering excessive inflammation and changing the structure of the extracellular matrix [12]. The tissue damage that follows chemical burns becomes worse because of the excessive reactive oxygen species production, which outstrips the ability of antioxidant defence systems to protect against damage while creating conditions that activate angiogenic signalling pathways [1,13].

The study excluded aflibercept and pegaptanib because their wider ligand-binding profiles and unique molecular structures would create confusion about redox results and hinder direct mechanistic evaluation. Consequently, bevacizumab and ranibizumab were selected as the focal agents for this study due to their widespread clinical use in ophthalmology, their well-characterised and distinct pharmacokinetic profiles (full-length antibody versus Fab fragment), and their established role as the primary comparators in translational anti-VEGF research, thereby providing a clear and direct framework to assess agent-specific effects on redox balance [10].

Clinical studies have not yet established how ranibizumab and bevacizumab differentially affect redox homeostasis in both the cornea and the rest of the body [8,14]. The researchers studied how ranibizumab and bevacizumab, which are administered through subconjunctival injections, affected oxidative stress markers in rabbits with alkali burn-induced corneal neovascularisation [2,13]. The study aims to show potential translational value for clinical CNV management through its examination of total oxidant status (TOS), total antioxidant status (TAS), and oxidative stress index (OSI) in corneal tissue and plasma and erythrocytes [15,16].

## 2. Materials and Methods

### 2.1. Animals and Ethical Approval

The research team studied 24 male New Zealand White rabbits who had ages between 10 and 12 months old and weight ranges of 2.5 to 3.0 kg. The laboratory maintained a climate-controlled environment for the rabbits, which kept a temperature of 20 ± 1 °C and a 12 h light/dark cycle. The animals had unrestricted access to both standard chow and drinking water. The study used six animals per group as a sample size because researchers needed to follow ethical approval regulations and the 3R principles (Replacement, Reduction, Refinement) for animal research. The experimental procedures of the study operated in accordance with all applicable regulations, which researchers established through the ARVO Statement for the Use of Animals in Ophthalmic and Vision Research. All experimental procedures were approved by the Local Ethics Committee of Kafkas University (Approval No: 2011/002, approved on 25 March 2011) and conducted in accordance with EU Directive 2010/63/EU.

### 2.2. Experimental Design and Group Allocation

Animals underwent a 1-week acclimatisation phase before they received their experimental assignments based on computer-generated randomisation, which divided them into four experimental groups that included six animals per group:

- Healthy control group 1 (HC) received no treatment.
- Group 2 (C): The treatment involved combined alkali injury with subsequent subconjunctival saline administration.
- Group 3 (BV): The treatment involved administering 0.5 mg/0.02 mL of bevacizumab (0.5 mg/0.02 mL) for the treatment of alkali injury through subconjunctival delivery.
- Group 4 (RN): The treatment involved administering 0.5 mg/0.02 mL of ranibizumab (0.5 mg/0.02 mL) for the treatment of alkali injury through subconjunctival delivery.

The researchers monitored all animals during the 14 days following their injuries before conducting biological sample collection and animal euthanasia.

### 2.3. Alkali Injury Induction and Anaesthesia

The process of general anaesthesia was performed through the injection of ketamine hydrochloride at a dosage of 35 mg/kg and xylazine hydrochloride at a dosage of 5 mg/kg. The application of 0.5% proparacaine hydrochloride (Alcaine^®^; Alcon Laboratories, Inc., Fort Worth, TX, USA) as a topical anaesthetic occurred one minute before the induction of the injury. The modified silver nitrate stick with 75% silver nitrate and 25% potassium nitrate was used to create an alkali injury by applying it to the central cornea of the right eye for 2 s. The left eyes remained untreated to function as internal control subjects for the experiment. The animals received two daily applications of topical antibiotic ointment (Tobrased^®^, Bilim Pharmaceuticals, Istanbul, Turkey) throughout a period of 7 days. The study period showed no occurrence of corneal perforation or necrosis in any of the animals.

### 2.4. Treatment Administration

The medical procedure involved a single subconjunctival injection, which took place 24 h after the patient sustained an alkali injury to the superotemporal bulbar conjunctiva at a distance of 3 to 4 mm from the limbus through a 30-gauge insulin syringe. All alkali injury inductions and subconjunctival injections required the same experienced ophthalmologist to perform them because this approach reduced inter-operator variability. Control group animals received normal saline treatment, while treatment groups received bevacizumab at 0.5 mg or ranibizumab at 0.5 mg as their respective treatments. The experienced ophthalmologist conducted all necessary procedures for alkali injury induction and subconjunctival injection, while physician investigators assisted with their duties to handle animals and provide care during the peri-procedural time.

### 2.5. Sample Collection and Processing

The animals were euthanised on day 14 after their injuries using sevoflurane anaesthesia, which had a 5% induction and 2.5% maintenance dose. Medical staff collected blood samples through cardiac puncture to obtain 5–7 mL of blood, which they stored in EDTA-containing tubes before performing enucleation. The researchers cut corneal tissues at the limbal boundary with Vannas scissors before they rinsed the samples in ice-cold phosphate-buffered saline (PBS; pH 7.4) and snap-froze them in liquid nitrogen to keep them until they would be analysed at −80 °C. The centrifugation process separated plasma from blood components by spinning at 2000× *g* for 15 min at 4 degrees Celsius. The researchers washed erythrocytes three times with cold saline solution before they used distilled water to destroy the cells. The researchers kept plasma and erythrocyte lysates at −80 °C until they needed to conduct biochemical tests.

### 2.6. Biochemical Analysis

The researchers used 1 mL of ice-cold phosphate-buffered saline (PBS) at pH 7.4 to homogenise corneal tissues before they started the centrifugation process, which lasted for 10 min at 4 °C with a force of 10,000× *g*. The researchers used supernatant liquid to perform TAS and TOS testing. The researchers determined TAS and TOS levels through the use of commercial colourimetric assay kits, which contained different products for TAS and TOS testing according to the Erel method (Rel Assay Diagnostics, Gaziantep, Turkey; Catalogue No: E-BC-K801-M for TAS and Catalogue No: E-BC-K802-M for TOS). Researchers express TAS values in millimoles of Trolox equivalent per litre (mmol Trolox equiv./L) and TOS values in micromoles of hydrogen peroxide equivalent per litre (μmol H_2_O_2_ equiv./L). The researchers conducted all assays twice while keeping the intra-assay and inter-assay coefficients of variation below 5%.

### 2.7. Sample Size Justification and Post Hoc Power Analysis

A formal a priori sample size calculation to achieve 95% confidence (α = 0.05) for the oxidative stress endpoints was not feasible before the study, as preliminary data on expected effect sizes (e.g., mean differences and variability in TAS/TOS) in this specific alkali-injury model treated with anti-VEGF agents were unavailable. Therefore, the group size of *n* = 6 was determined pragmatically, considering the 3R principles (Reduction), ethical approval limitations, resource availability, and alignment with group sizes commonly used in comparable experimental corneal neovascularisation research [2]. The researchers conducted a post hoc power analysis to test the statistical strength of their results, which they measured through all oxidative stress parameters using effect sizes, with α set at 0.05 (Table S1). The study demonstrated sufficient power to identify large effects that the researchers had found in major corneal TOS and plasma TOS measurements. The study achieved a power of 62 per cent for blood TOS analysis because the power needed to identify actual differences, which the researchers had expected to see, remained above that threshold. The inter-group differences in systemic redox markers found no statistically significant results; therefore, researchers should treat this data as early findings that need larger validation studies with specific power to confirm results.

## 3. Results

The table shows TAS and TOS measurement results from plasma, blood erythrocytes, and corneal tissues for all four experimental groups. The statistical tests confirmed that different groups had significant differences in their oxidative and antioxidant measurements, which were observed in plasma and blood and corneal tissue samples.

### 3.1. Corneal Tissue Oxidiser/Antioxidant Status

**Figure 1:**
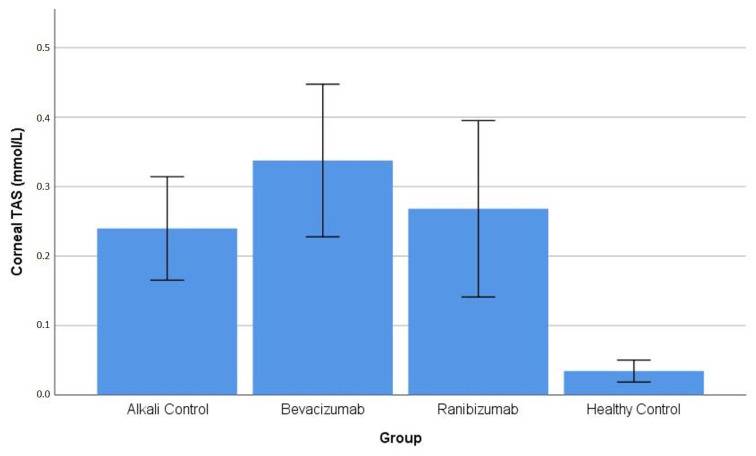
Corneal TAS levels in healthy control and alkali-injured groups. All alkali-injured groups showed significantly increased corneal TAS levels compared with the healthy control group, with no statistically significant differences among the alkali-injured groups (Table 1).

Corneal TAS: The Kruskal–Wallis test results showed that corneal TAS measurements differed among the experimental groups, which resulted in null hypothesis rejection (*p* = 0.002). The post hoc Dunn–Bonferroni pairwise comparisons showed that the HC group had corneal TAS levels which were significantly lower in comparison to the AC group (*p* = 0.015), the BV group (*p* = 0.007), and the RN group (*p* < 0.001). The alkali-injured groups (AC, BV, RN) showed no significant differences when each group was compared to the others (all *p* > 0.05) (Table 1). The distribution of corneal TAS values across the experimental groups is illustrated in Figure 1.

**Figure 2:**
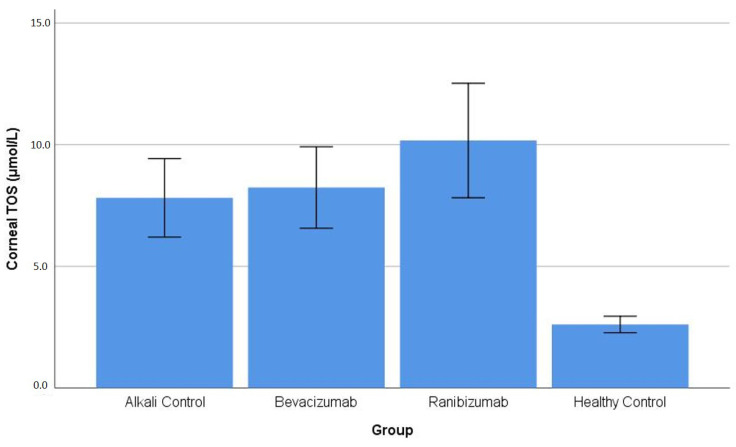
Corneal TOS levels in healthy control (HC) and alkali-injured experimental groups. Values are presented as mean ± SD. Corneal TOS levels were significantly higher in all alkali-injured groups compared with the healthy control group, while no statistically significant differences were observed among the alkali-injured groups (Table 1).

Corneal TOS: The statistical analysis showed that corneal TOS levels under study exhibited different results among the groups that were tested (*p* < 0.001). The HC group showed the lowest corneal TOS values, which were recorded as 2.61 ± 0.34 μmol/L, according to expectations. The AC group showed a marked increase in corneal TOS, which exceeded the levels of the HC group, and the rest of the alkali-injured groups showed similar results, with the BV group showing 8.24 ± 1.67 μmol/L and the RN group showing 10.17 ± 2.35 μmol/L (all *p* < 0.001 vs. HC). The Bonferroni post hoc analysis showed that the alkali-injured groups (AC, BV, RN) had no significant differences in their corneal TOS levels (RN vs. AC: *p* = 0.141; RN vs. BV: *p* = 0.349; AC vs. BV: *p* = 1.000) (Table 1). The distribution of corneal TOS values across the experimental groups is illustrated in Figure 2.

### 3.2. Oxidant/Antioxidant Status in Blood (Erythrocytes)

**Figure 3:**
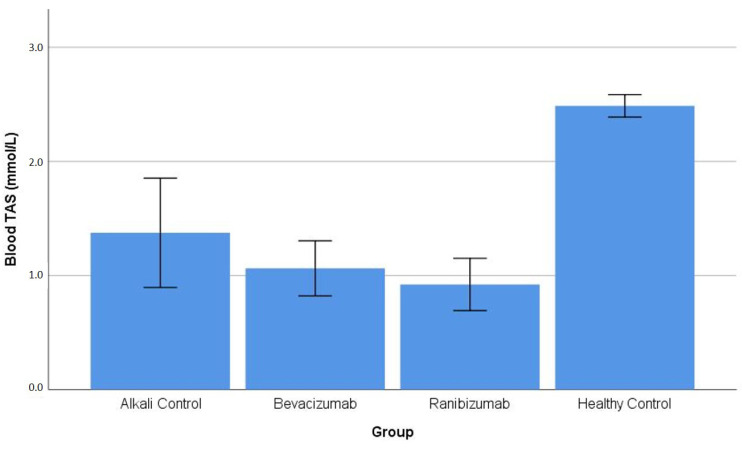
Blood (erythrocyte) total antioxidant status (TAS) levels across experimental groups. The healthy control group exhibited significantly higher erythrocyte TAS levels compared with all alkali-injured groups. No significant differences were detected among the injured groups (Table 1).

Blood TAS: The experimental groups showed substantial differences in blood total antioxidant status because their test results had a *p*-value below 0.001. The HC group exhibited significantly higher erythrocyte TAS values (2.49 mmol/L) compared with all alkali-injured groups. The injured groups all showed blood TAS levels that were significantly lower than the HC group, which included AC (1.37 ± 0.48 mmol/L), BV (1.06 ± 0.24 mmol/L), and RN (0.92 ± 0.23 mmol/L) (all *p* < 0.008 vs. HC). The injured groups showed no significant differences between them because AC vs. BV (*p* = 0.524) and AC vs. RN (*p* = 0.243) (Table 1) showed no statistically significant difference. The distribution of blood TAS values across the experimental groups is illustrated in Figure 3.

**Figure 4:**
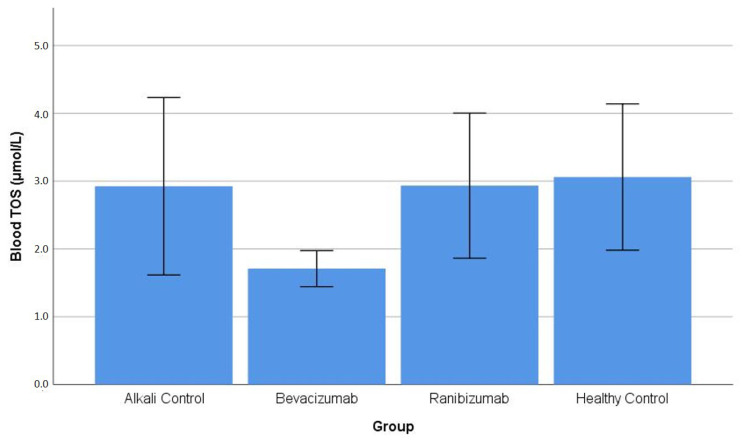
Blood (erythrocyte) total oxidant status (TOS) levels across experimental groups. No statistically significant differences in blood TOS levels were detected among the groups, as confirmed by one-way ANOVA and post hoc analysis (Table 1).

Blood TOS: One-way ANOVA revealed no statistically significant differences in blood total oxidant status among the experimental groups (*p* = 0.102). Games–Howell post hoc comparisons confirmed the absence of significant pairwise differences, including BV vs. AC (*p* = 0.227) and all other group comparisons (*p* ≥ 0.094) (Table 1). The distribution of blood TOS values across the experimental groups is illustrated in Figure 4.

### 3.3. Oxidative/Antioxidative Status in Plasma

**Figure 5:**
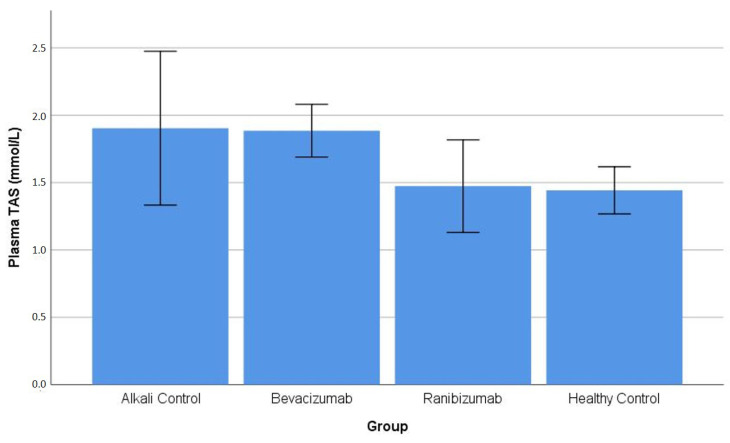
Plasma total antioxidant status (TAS) levels across experimental groups. Plasma TAS values were lower in alkali-injured groups compared with the healthy control group; however, no statistically significant differences were observed among the experimental groups (Table 1).

Plasma TAS: The one-way ANOVA test showed no significant differences in plasma total antioxidant status between the different groups (*p* value 0.056). The Games–Howell post hoc analysis showed no significant differences between any of the tested pairs (all *p* values 0.126 or higher) (Table 1). The distribution of plasma TAS values across the experimental groups is illustrated in Figure 5.

**Figure 6:**
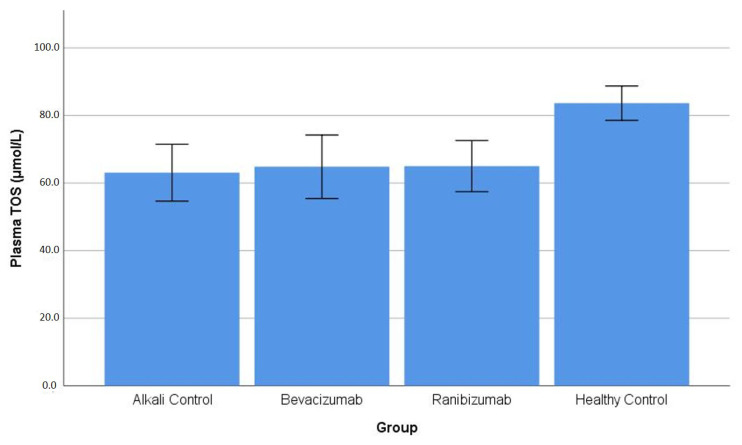
Blood (erythrocyte) total oxidant status (TOS) levels across experimental groups. No statistically significant differences in blood TOS levels were detected among the groups, as confirmed by one-way ANOVA and post hoc analysis (Table 1).

Plasma TOS: The study found that total oxidant status in plasma showed a substantial group effect, which reached a significance level below 0.001. The healthy control group showed the highest plasma TOS levels at 83.64 ± 5.10 μmol/L, which exceeded the levels found in all alkali-injured groups, including AC (63.08 ± 8.41 μmol/L; *p* = 0.001), BV (64.83 ± 9.39 μmol/L; *p* = 0.003), and RN (65.02 ± 7.57 μmol/L; *p* = 0.003). The study showed that plasma TOS levels remained constant among all alkali-injured groups since the *p*-values reached 1.000 or higher (Table 1). The distribution of plasma TOS values across the experimental groups is illustrated in Figure 6.

### 3.4. Macroscopic Findings

**Figure 7:**
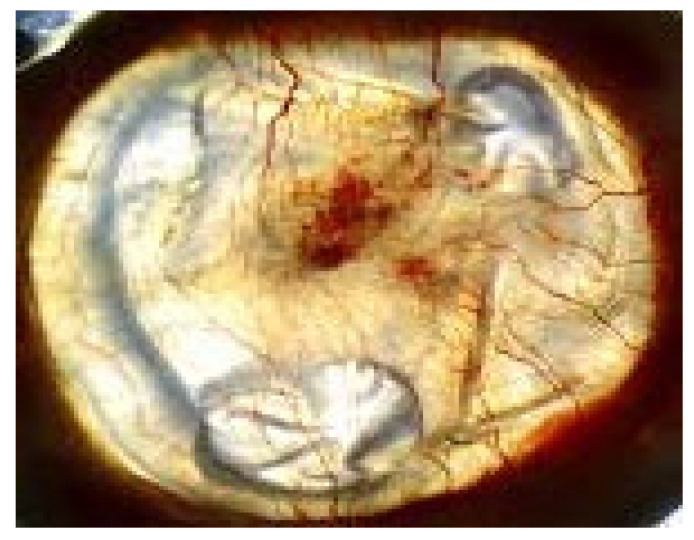
Representative macroscopic corneal photograph of the healthy control (HC) group on day 14.

**Figure 8:**
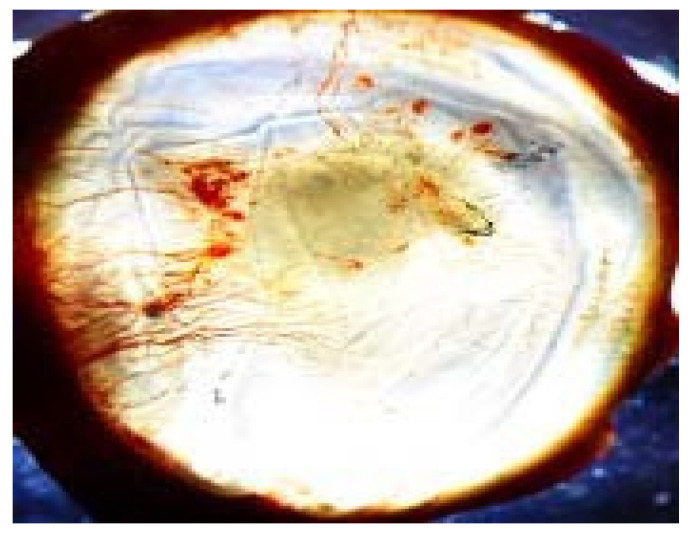
Representative macroscopic corneal photograph of the bevacizumab-treated (BV) group on day 14.

**Figure 9:**
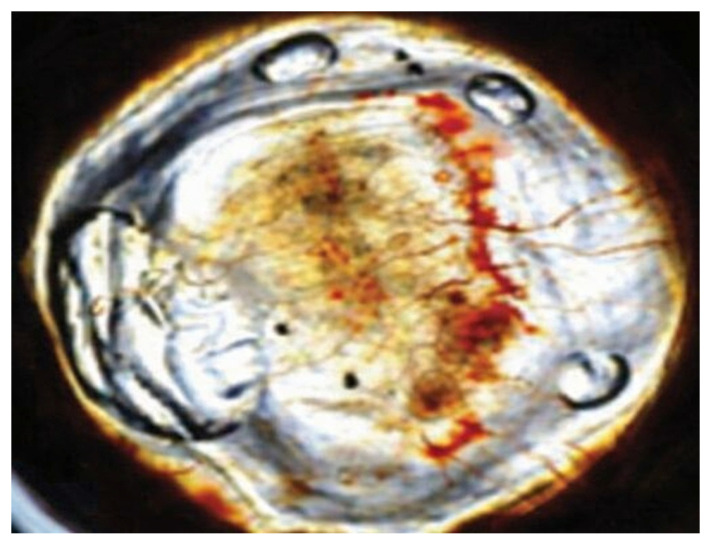
Representative macroscopic corneal photograph of the ranibizumab-treated (RZB) group on day 14.

The experimental groups’ corneal photographs shown in Figure 7, Figure 8 and Figure 9 present typical macroscopic images of the cornea. The images serve to show the corneal surface changes which occurred after alkali injury treatment and after anti-VEGF treatment yet they do not provide any quantitative data or statistical results.

## 4. Discussion

The current research compares how alkali corneal injuries affect oxidant and antioxidant levels through anti-VEGF treatment in both corneas and systemic body areas. The study results show that oxidative stress serves as a central factor in developing alkali-induced corneal neovascularisation (CNV), while different agents produced particular redox changes after administering bevacizumab and ranibizumab, which matched new pharmacokinetic research and ocular experimental model data about tissue penetration and drug distribution [17,18,19,20,21].

The biochemical data particularly show increased corneal TOS levels, providing a biochemical context for oxidative stress following alkali injury. These findings are consistent with the severe tissue damage induced by chemical burns and should be interpreted independently of previously reported morphological observations from similar experimental models. All alkali-injured groups demonstrated elevated corneal TOS levels, reflecting intense local oxidative stress associated with neutrophil infiltration and increased reactive oxygen species (ROS) production, leading to epithelial and stromal injury [3,22,23]. All injured groups showed higher corneal TAS levels when compared to healthy controls, which indicates that the body produces more antioxidants to protect itself from oxidative damage. The continuous high TOS levels caused an overall prooxidant shift, which demonstrated that the response to oxidative stress remained below required levels.

The study shows that different anti-VEGF agents produce different results in their effects on the redness of the cornea. The administration of ranibizumab was associated with numerically higher corneal TOS values compared with both the alkali control and bevacizumab-treated groups; however, these differences did not reach statistical significance (*p* = 0.141 vs. AC; *p* = 0.349 vs. BV). Accordingly, this observation should be interpreted as a descriptive trend rather than evidence of a definitive or superior pro-oxidant effect. Ranibizumab exerts its pharmacological effect through high-affinity VEGF-A inhibition mediated by its Fab fragment structure, which facilitates corneal tissue penetration. It has been suggested in the literature that VEGF may have cytoprotective and redox-modulating roles beyond angiogenesis; however, such mechanisms were not directly assessed in the present study. Therefore, any potential association between VEGF neutralisation and local redox imbalance remains speculative and should be interpreted as a hypothesis-generating observation rather than a demonstrated mechanism. The finding requires cautious interpretation because direct mechanistic measurements are missing, and it should be treated as an associative biochemical observation that does not prove causation. Prior research has shown that ranibizumab penetrates the cornea better than larger anti-VEGF molecules [2,13], which might explain why different local tissues show different exposure levels. The available research data currently does not establish any direct relationship between VEGF inhibition, tissue penetration, and redox modulation [19,23]. The current study provides evidence that supports earlier research findings that ranibizumab penetrates the cornea better than larger anti-VEGF molecules [2,13,18].

The drug ranibizumab works by blocking VEGF-A through its Fab fragment structure, which enables the drug to penetrate corneal tissues, as shown in previous studies. Previous studies through experimental and theoretical methods have shown that endothelial and epithelial cells under stress conditions use VEGF for cytoprotective purposes, while it also serves its primary function of promoting angiogenesis. The study did not evaluate those effects that exist beyond the angiogenic properties of VEGF. After VEGF neutralisation, the body may experience changes to its local redox balance, which scientists currently consider a theoretical possibility rather than a proven fact [24]. The researchers showed reduced VEGF expression after ranibizumab treatment in previous studies, which used immunohistochemical methods. The study conducted direct VEGF measurements and immunohistochemical analyses to confirm these results. The researchers established that corneal oxidative parameters changed because of factors other than VEGF suppression. The elevated corneal oxidative stress levels in the ranibizumab-treated group should be understood as a biochemical association rather than proof of either stronger anti-angiogenic effects or increased pro-oxidant activity.

Bevacizumab produced a moderate impact on corneal redox status because its TOS values showed higher results than those of normal controls, yet matched the findings of the alkali control group with a *p*-value of 1.000. The larger molecular size of bevacizumab and its full-length IgG structure likely limits corneal penetration, which leads to decreased local VEGF signalling control and results in reduced impact on corneal oxidative balance. The interpretation supports the finding that smaller anti-VEGF molecules deliver better to the anterior segment of the eye than larger molecules, according to the results of delivery studies that compare different anti-VEGF molecules [24,25].

All alkali-injured groups showed erythrocyte TAS depletion at the systemic level because their levels dropped significantly below those of healthy controls, which indicated that severe local injury had triggered major antioxidant consumption or redistribution. The two anti-VEGF agents did not produce significant reductions beyond what the alkali control group showed, yet the observed trends should be studied because they may influence the system’s redox status during off-label anti-VEGF treatment.

The research found that bevacizumab treatment resulted in reduced blood TOS levels when compared to the alkali control group; however, this difference did not reach statistical significance (*p* = 0.227). Bevacizumab-treated animals showed a non-significant tendency toward lower systemic TOS levels compared with the alkali control group. While the prolonged systemic half-life of bevacizumab due to Fc-mediated recycling has been described, the present findings do not provide direct evidence for a causal relationship between bevacizumab exposure and systemic redox modulation. The drug maintains its active presence in the circulation because its Fc region enables recycling and prolonged systemic exposure [6]. Accordingly, the observed systemic redox changes should be interpreted cautiously and considered hypothesis-generating, requiring further investigation in studies incorporating longitudinal sampling and repeated dosing protocols [8,26,27].

Ranibizumab demonstrates a complete absence of the Fc region, which leads to its rapid removal from the bloodstream, resulting in a reduced impact on the body’s redox balance.

The study discovered that healthy controls had higher plasma TOS levels compared to injured groups at all levels of injury (*p* < 0.001). The body produces oxidants through its normal metabolic processes, which creates this physiological oxidant production pattern. The researchers believe that severe alkali injury activates an acute-phase response, which leads to the body redistributing and using or storing its oxidant resources, thus decreasing plasma TOS measurements. The study discovered that systemic redox balance undergoes active control through different body compartments, which requires continuous studies to examine redox changes that occur over time [10].

The research shows that ranibizumab and bevacizumab produce different effects on the body’s redox balance in both local and systemic areas of the body [28,29,30]. The study found that ranibizumab produced the greatest corneal TOS results, which probably indicates strong local anti-angiogenic effects, while bevacizumab showed a pattern of reducing overall oxidant levels and producing less potent local results. The two agents produce different redox responses in local and systemic areas of the body according to the observed results [24].

### Clinical Implications

- The current results require careful interpretation because the study failed to find any statistically significant differences between the anti-VEGF-treated groups in their main oxidative stress assessments. The data do not provide evidence that any specific treatment brings better results than others or produces specific clinical benefits.
- The local and systemic trends in redox parameters should be treated as exploratory findings that create new research possibilities. The studied substances show different metabolic characteristics, which cause varying results but lead to no biologically active effects or therapeutic success. The observed patterns should not be understood as proof that either agent achieves better results while damaging redox functions.
- The research shows that oxidative stress in corneal neovascularisation develops through injury-related mechanisms, while any differences between agents occur at levels that do not reach statistical significance for this study.
- Future studies need to validate these exploratory biochemical patterns because they require larger sample sizes and longitudinal redox measurements that must be correlated with clinical and histopathological outcomes.

## 5. Limitations

The study results require consideration of multiple limitations that affect their interpretation.

The TAS and TOS assays enable researchers to assess the overall redox status of samples, but they fail to identify specific reactive oxygen species and particular antioxidant defence mechanisms. The researchers could not discover the specific molecular elements that caused the redox changes they saw. Redox modulation studies would benefit from future research that examines enzymatic antioxidants (such as SOD, catalase, and glutathione peroxidase) together with lipid peroxidation products and nitrosative stress markers [23]. The research team chose to study only male rabbits because it reduced hormonal differences, but this decision created challenges for studying female rabbits. The study design failed to evaluate potential variations between male and female participants because sex hormones control pathways related to inflammation, blood vessel formation, and oxidative stress treatment. The research team gathered redox data at a single time point on day 14 after the injury, which prevented them from studying how redox levels changed during the acute and subacute phases following alkali injury and anti-VEGF treatment. The researchers needed to conduct longitudinal sampling to determine whether the redox patterns they observed represented short-term adaptive changes or permanent biological modifications [10]. An additional important limitation is the lack of baseline redox measurements. The researchers evaluated blood and corneal redox status at the study’s conclusion on day 14 because all animals lacked pre-injury and pre-treatment measurements. The study establishes group differences at the final measurement point but fails to determine individual rabbit changes from their initial baseline. This design precludes a true longitudinal analysis within subjects and means that the reported differences could be influenced by unaccounted-for baseline variability between animals, rather than being solely a consequence of the alkali injury or the anti-VEGF treatments. The researchers assessed biochemical changes through previous research, which documented morphological results, but they found no connection between redox values and specific corneal neovascularisation measurements or histopathological evaluations within the same study group. The scientific community would find stronger evidence from biochemical studies if researchers found links between specific metabolic changes and particular histopathological outcomes. The researchers used a single-dosage method to deliver anti-VEGF agents through a single route, which involved administering the medication via subconjunctival injection. The study design hinders direct comparison to actual clinical settings because it uses one delivery method and one dosage method, which does not match the common medical practice of using multiple dosing methods and different administration techniques. The study found that post hoc power analysis showed adequate power for the main study objectives, but the small sample size created difficulties in identifying minor differences between the intervention groups with respect to systemic redox measurements. The study results demonstrate that larger studies are required to confirm the findings of this research. The alkali burn model fails to reproduce the complete range of human corneal neovascularisation, which develops during chronic inflammation that accompanies pre-existing eye surface conditions. The study establishes clear local and systemic redox effects that occur due to two commonly prescribed anti-VEGF drugs. The study’s limitations create a precise path for researchers to follow during future studies, which will explore the underlying mechanisms and practical applications of the research findings.

## 6. Conclusions

The research study shows that alkali-induced corneal neovascularisation causes significant changes to both local tissues and systemic compartments that disrupt redox equilibrium. The different experimental groups showed distinct biochemical reactions to anti-VEGF treatment, which created separate redox patterns for each treatment group. Ranibizumab treatment resulted in higher corneal oxidative stress values compared to other alkali-injured groups, although the differences between these groups did not reach statistical significance, which requires careful interpretation. Bevacizumab exhibited a tendency to decrease systemic oxidant levels, which did not achieve statistical significance between the two anti-VEGF treatment groups. The results indicate that anti-VEGF agents produce different biochemical effects because of their specific pharmacological and pharmacokinetic properties, which do not provide evidence for either therapeutic superiority or causal relationships. The research study offers biochemical information about anti-angiogenic treatment by studying how local and systemic redox patterns function in an experimental corneal neovascularisation model. The biological and clinical importance of redox changes will be understood through future research, which includes continuous redox observation and direct neovascularisation assessment and enhanced delivery methods, while researchers seek to optimise anti-VEGF therapy after chemical corneal injury.

## Figures and Tables

**Table 1:** TAS and TOS levels in plasma, blood (erythrocytes), and corneal tissues across experimental groups.

	Alkali Control	Bevacizumab	Ranibizumab	Healthy Control	*p*
	Mean ± SD or Median (Q1–Q3)	Mean ± SD or Median (Q1–Q3)	Mean ± SD or Median (Q1–Q3)	Mean ± SD or Median (Q1–Q3)	
Plasma TAS (mmol/L)	1.904 ± 0.571	1.885 ± 0.196	1.473 ± 0.344	1.443 ± 0.175	0.056
Blood TAS (mmol/L)	1.374 ± 0.479	1.063 ± 0.241	0.922 ± 0.229	2.487 ± 0.099	<0.001
Corneal TAS (mmol/L)	0.23 (0.19–0.31)	0.36 (0.29–0.42)	0.29 (0.12–0.38)	0.04 (0.039–0.042)	0.002
Plasma TOS (μmol/L)	63.078 ± 8.411	64.834 ± 9.392	65.021 ± 7.567	83.641 ± 5.104	<0.001
Blood TOS (μmol/L)	2.924 ± 1.309	1.709 ± 0.266	2.934 ± 1.07	3.061 ± 1.078	0.102
Corneal TOS (μmol/L)	7.815 ± 1.613	8.241 ± 1.673	10.17 ± 2.353	2.611 ± 0.339	<0.001

Notes: Data are presented as mean ± SD or median (interquartile range). The Kruskal–Wallis test with Dunn’s post hoc test was used for corneal TAS. One-way ANOVA followed by the Games–Howell post hoc test was used for plasma TAS, blood TAS, and blood TOS, while the Bonferroni post hoc test was used for plasma TOS and corneal TOS.

## Data Availability

The current study’s animal experimental data remains inaccessible to the public because of ethical and institutional restrictions that govern its release. The corresponding author will provide data to anyone who requests it through a legitimate process.

## Supplementary Materials

The following supporting information can be downloaded at: https://www.mdpi.com/article/10.3390/life16030488/s1, Table S1: Power Analysis for Primary and Secondary Endpoints in the Rabbit Corneal Neovascularization Model (G*Power Calculations, α = 0.05).

## References

[1] Shoham A., Hadziahmetovic M., Dunaief J.L., Mydlarski M.B., Schipper H.M. (2008). Oxidative stress in diseases of the human cornea. Free Radic. Biol. Med..

[2] Ekinci M., Ulviye Yiğit F., Oba M.E., Çağatay H.H., Hüseyinoğlu Ü., Yakan S., Arslan B. (2011). Inhibition of Corneal Neovascularization by Ranibizumab (Lucentis): An Experimental Study in Rabbit Cornea. Kafkas Univ. Vet. Fak. Derg..

[3] Adamis A.P., Shima D.T. (2005). The role of vascular endothelial growth factor in ocular health and disease. Retina.

[4] Gakhramanov F.S. (2005). Effect of natural antioxidants on antioxidant activity and lipid peroxidation in eye tissue of rabbits with chemical burns. Bull. Exp. Biol. Med..

[5] Phillips G.D., Stone A.M., Jones B.D., Schultz J.C., Whitehead R.A., Knighton D.R. (1994). Vascular endothelial growth factor (rhVEGF165) stimulates direct angiogenesis in the rabbit cornea. In Vivo.

[6] Toto L., Di Antonio L., Costantino O., Mastropasqua R. (2021). Anti-VEGF therapy in myopic CNV. Curr. Drug Targets.

[7] Lien S., Lowman H.B. (2008). Therapeutic anti-VEGF antibodies. Therapeutic Antibodies.

[8] Wang J., Kunikata H., Yasuda M., Himori N., Nitta F., Nakazawa T. (2024). Systemic Oxidative Stress Level as a Pathological and Prognostic Marker in Myopic Choroidal Neovascularization. Ophthalmol. Sci..

[9] Khachigian L.M., Liew G., Teo K.Y., Wong T.Y., Mitchell P. (2023). Emerging therapeutic strategies for unmet need in neovascular age-related macular degeneration. J. Transl. Med..

[10] Kuroki M., Voest E.E., Amano S., Beerepoot L.V., Takashima S., Tolentino M., Kim R.Y., Rohan R.M., Colby K.A., Yeo K.T. (1996). Reactive oxygen intermediates increase vascular endothelial growth factor expression in vitro and in vivo. J. Clin. Investig..

[11] Colavitti R., Pani G., Bedogni B., Anzevino R., Borrello S., Waltenberger J. (2002). Reactive oxygen species as downstream mediators of angiogenic signaling by vascular endothelial growth factor receptor-2/KDR. J. Biol. Chem..

[12] Jung J.H., Kim S.S., Chung H., Hejri A., Prausnitz M.R. (2022). Six-month sustained delivery of anti-VEGF from in-situ forming hydrogel in the suprachoroidal space. J. Control. Release.

[13] Ekinci M., Çağatay H.H., Yazar Z., Bingöl S.A., Kaplan A. (2013). Inhibition of Corneal Neovascularization by Subconjunctival Injection of Ranibizumab and Bevacizumab in Rabbit Cornea. Kafkas Univ. Vet. Fak. Derg..

[14] Wu D., Chan K.E., Lim B.X.H., Lim D.K.A., Wong W.M., Chai C., Lim C.H.L. (2024). Management of corneal neovascularization: Current and emerging therapeutic approaches. Indian J. Ophthalmol..

[15] Erçin Akıdan E., Yılmaz E., Yılmaz N., Akıdan M. (2024). Increased oxidative stress biomarkers in central serous chorioretinopathy. Sci. Rep..

[16] Kurutas E.B. (2015). The importance of antioxidants which play the role in cellular response against oxidative/nitrosative stress: Current state. Nutr. J..

[17] Avery R.L., Castellarin A.A., Steinle N.C., Dhoot D.S., Pieramici D.J., See R., Couvillion S., Nasir M.A., Rabena M.D., Le K. (2014). Systemic pharmacokinetics following intravitreal injections of ranibizumab, bevacizumab or aflibercept in patients with neovascular AMD. Br. J. Ophthalmol..

[18] Eski M.T., Teberik K., Oltulu P., Ankaralı H., Kaya M., Alpay M. (2022). The effects of subconjunctival bevacizumab, ranibizumab, and aflibercept on corneal neovascularization. Hum. Exp. Toxicol..

[19] Al-Debasi T., Al-Bekairy A., Al-Katheri A., Al Harbi S., Mansour M. (2017). Topical versus subconjunctival anti-vascular endothelial growth factor therapy (Bevacizumab, Ranibizumab and Aflibercept) for treatment of corneal neovascularization. Saudi J. Ophthalmol..

[20] Zhu Q., Ziemssen F., Henke-Fahle S., Tatar O., Szurman P. (2008). Aisenbrey, Vitreous levels of bevacizumab and vascular endothelial growth factor-A in patients with choroidal neovascularization. Ophthalmology.

[21] Freitas L.G., Isaac D.L., Tannure W.T., Gabriel L.A., Reis R.G., Rassi A.R. (2013). Intravitreal bevacizumab combined with infliximab in the treatment of choroidal neovascularization secondary to age-related macular degeneration: Case report series. Arq. Bras. Oftalmol..

[22] Cejka C., Cejkova J. (2015). Oxidative stress to the cornea, changes in corneal optical properties, and advances in treatment of corneal oxidative injuries. Oxid. Med. Cell. Longev..

[23] Ahmad A., Ahsan H. (2020). Biomarkers of inflammation and oxidative stress in ophthalmic disorders. J. Immunoass. Immunochem..

[24] Nethi S.K., Barui A.K., Mukherjee S., Patra C.R. (2019). Engineered nanoparticles for effective redox signaling during angiogenic and antiangiogenic therapy. Antioxid. Redox Signal..

[25] Cheng R., Li C., Li C., Wei L., Li L., Zhang Y., Yao Y., Gu X., Cai W., Yang Z. (2013). The artemisinin derivative artesunate inhibits corneal neovascularization by inducing ROS-dependent apoptosis in vascular endothelial cells. Investig. Ophthalmol. Vis. Sci..

[26] Ng D.S., Ho M., Iu L.P., Lai T.Y. (2022). Safety review of anti-VEGF therapy in patients with myopic choroidal neovascularization. Expert Opin. Drug Saf..

[27] Pożarowska D., Pożarowski P. (2016). The era of anti-vascular endothelial growth factor (VEGF) drugs in ophthalmology, VEGF and anti-VEGF therapy. Cent. Eur. J Immunol..

[28] Gan L., Fagerholm P., Palmblad J. (2004). Vascular endothelial growth factor (VEGF) and its receptor VEGFR-2 in the regulation of corneal neovascularization and wound healing. Acta Ophthalmol. Scand..

[29] Manzano R.P., Peyman G.A., Khan P., Carvounis P.E., Kivilcim M., Ren M. (2007). Inhibition of experimental corneal neovascularisation by bevacizumab (Avastin). Br. J. Ophthalmol..

[30] Murata M., Shimizu S., Horiuchi S., Taira M. (2006). Inhibitory effect of triamcinolone acetonide on corneal neovascularization. Graefes Arch. Clin. Exp..

