# Supplementary material for: Effects of Subconjunctival Bevacizumab and Ranibizumab on Corneal and Systemic Oxidative Stress Biomarkers in an Alkali Injury Model
Source: Life (Basel). 2026 Mar 17;16(3):488. doi: 10.3390/life16030488 (PMC13027824; doi:10.3390/life16030488)
Supplement: Supplementary file 1 [file life-16-00488-s001.zip › life-4084482-supplementary.pdf]

Table S1. Power Analysis for Primary and Secondary Endpoints in the Rabbit Corneal Neovascularization Model (G*Power Calculations, α=0.05).

Parameter	Cohen's f (Effect Size)	Strength (%)	Explanation (Article Context)	n/group Required for 80% Power
**Plasma TAS** (p=0.056, trend)	0.676 (medium-large)	71.2	Subtile group difference; NS but strong trend	12
**Blood TAS** (p<0.001)	0.905 (large)	93.5	Strong difference (injured < HC); well powered	5
**Plasma TOS** (p<0.001)	0.874 (large)	91.7	Unexpected HC > injured; high force	6
**Blood TOS** (p=0.102, NS)	0.616 (medium-large)	62.3	Between-group NS; underpowered, suggest equivalence testing	10
**Corneal TOS** (p<0.001)	0.873 (large)	91.7	RN trend ↑ (pro-oxidant); significant, well powered	6
**Corneal TAS** (approx., p=0.002)	0.705 (medium-large)	75.2	HC < injured; non-parametric but strong	11

Note: The study has sufficient strength to detect large effects which have statistical values (blood/plasma TAS/TOS) (f 0.6) and it fails to detect the smaller effects which have statistical value (blood TOS f 0.616).
